# T1ρ MRI of healthy and fibrotic human livers at 1.5 T

**DOI:** 10.1186/s12967-015-0648-0

**Published:** 2015-09-08

**Authors:** Anup Singh, Damodar Reddy, Mohammad Haris, Kejia Cai, K. Rajender Reddy, Hari Hariharan, Ravinder Reddy

**Affiliations:** Department of Radiology, CMROI, University of Pennsylvania, Philadelphia, PA USA; Center for Biomedical Engineering, Indian Institute of Technology Delhi, Block-II, Room No. 389, Hauz Khas, Delhi, 110016 India; Research Branch, Sidra Medical and Research Center, Doha, Qatar; Department of Radiology, University of Illinois at Chicago, Chicago, IL USA; Division of Gastroenterology, Department of Medicine, University of Pennsylvania, Philadelphia, PA USA

**Keywords:** MRI, Spin lock, T1ρ, Liver fibrosis

## Abstract

**Background:**

Liver fibrosis is a public health problem worldwide. There is a need of noninvasive imaging based methods for better diagnosis of this disease. In the current study, we aim to evaluate the potential of T1ρ MRI technique in detecting and characterizing different grades of liver fibrosis in vivo in humans.

**Methods:**

Healthy subjects and patients with liver fibrosis were prospectively recruited for T1ρ MRI of liver on a 1.5 T MR scanner. Single slice T1ρ weighted images were acquired at different spin lock duration (0, 10, 20 and 30 ms) with spin lock amplitude of 500 Hz in a single breath-hold. Additionally, liver’s T1ρ images were acquired from five healthy subjects on the same day (n = 2) and different day (n = 2) sessions for test–retest study. Liver biopsy samples from patients were obtained and used to calculate the METAVIR score to define the stage of fibrosis and inflammation grade. T1ρ maps were generated followed by computation of mean and standard deviation (SD) values. Coefficient of variation (COV) of T1ρ values between two MRI scans was computed to determine reproducibility in liver. *T* test was used to compare T1ρ values between healthy and fibrotic liver. Pearson correlation was performed between stages of liver fibrosis and T1ρ values.

**Results:**

The mean (SD) T1ρ value among subject with healthy liver was 51.04 (3.06) ms. The COV of T1ρ values between two repetitions in the same day session was 0.83 ± 0.8 % and in different day session was 5.4 ± 2.7 %. T1ρ values in fibrotic liver were significantly higher compared to those of healthy liver (p < 0.05). A statically significant correlation between stages of fibrosis and T1ρ values was observed (r = 0.99, p < 0.05). Inflammation score for one patient was 2 and for remaining patients it was 1.

**Conclusions:**

Proposed T1ρ pulse sequence design and protocol enabled acquisition of a single slice T1ρ weighted images in a single breath-hold and hence mitigated breathing motion related artifacts. Preliminary results have shown the sensitivity of T1ρ values to changes induced by liver fibrosis, and may potentially be used as a clinical biomarker to delineate the stages of liver fibrosis. Further, studies on a large number of subjects are required to validate the observations of the current study. Nevertheless, T1ρ imaging can be easily setup on a clinical scanner to monitor the progression of liver fibrosis and to the evaluate efficacy of anti-fibrotic drugs.

## Background

Liver cirrhosis and liver cancer are significant health problems worldwide. Liver fibrosis, which is due to damage/insults by toxic metabolites and viral infections [1], leads to cirrhosis. Chronic hepatitis C, chronic hepatitis B, alcoholic liver disease (ALD) and nonalcoholic fatty liver disease (NALD) are the most common causes of fibrosis progression.

Liver fibrosis is the deposition of excess and abnormal extracellular matrix (ECM), known as scar, in the liver in response to a variety of chronic liver injuries [1]. These matrix proteins include collagens, fibronectin, and proteoglycans. The severity of fibrosis is categorized based on the organization of matrix deposition in the liver. Cirrhosis, for example, refers to the end-stage of fibrosis in which parenchymal nodules are surrounded by scar tissue. Assignment to a specific stage has prognostic value and is important in the management of individual patients as well as in trials of potential anti-fibrotic agents. Inflammation is another condition that occurs in response to liver injury. Detection of early fibrosis offers multiple benefits and also aids in assessing disease severity and treatment response. When identified early, liver fibrosis is treatable, even at the stage of cirrhosis by using antiviral agents for hepatitis C and B and also taking steps to limit alcohol consumption, overweight and reducing the incidence of type-2 diabetes mellitus.

Liver biopsy is considered as the gold standard for diagnosis of fibrosis. Biopsies, however, carry a risk of significant morbidity and mortality [2]. Moreover, biopsies are plagued with poor reproducibility and may misclassify up to one-third of cirrhotic livers [3]. The limitations of biopsies have proven to be significant practical and financial barriers in clinical care and clinical trials. Existing non-invasive diagnostic tests include a variety of serum tests and transient elastography [Fibroscan and magnetic resonance elastography (MRE)] [4–6]. Among imaging methods, MRE have shown some potential in diagnosis and staging of liver fibrosis. However, MRE requires additional hardware and software, consequently limiting their availability in most clinical settings. Double contrast MRI using gadolinium chelates and super paramagnetic iron oxides (SPIOs) was suggested to visualize liver fibrosis directly based on the hepatic texture alterations [7, 8]. Other MR-based techniques including diffusion-weighted imaging, MR spectroscopy and magnetization transfer (MT) imaging had also shown limited sensitivity, especially in the early and middle stages of fibrosis [6, 9–11]. Spin–lattice relaxation time (T1) and spin–spin relaxation time (T2) MRI mapping have been used to study liver fibrosis. However, the clinical utility of these techniques for staging liver fibrosis has yet to be established. Research and clinical studies are being carried out to evaluate the potential of these techniques for better diagnosis as well as clinical feasibility.

Spin lock relaxation time constant in rotating frame (T1ρ) MRI technique is another potential technique, which can be used for studying liver fibrosis. This technique has been mainly used for investigating changes during knee osteoarthritis [12, 13], myocardial infarction [14], Alzheimer disease [15]. Recent preclinical study [16] on rat liver have shown the potential of T1ρ MRI in staging liver fibrosis. Increase in T1ρ values with increase in the stage of fibrosis has been reported [16]. The exact mechanism behind T1ρ changes in the liver is not very clear. As such, excessive accumulation of ECM should reduce T1ρ values; however, increase in T1ρ values suggests that other factors like inflammation and change in exchange rate as well as correlation times might dominate T1ρ contrast in liver fibrosis. A few T1ρ MRI studies of human liver have been carried in clinical scanners [17–19]. These preliminary studies have also shown that fibrotic liver exhibits higher T1ρ values compared to normal liver. However, these clinical studies have investigated only late stage fibrosis patients. More T1ρ MRI studies are required, with different stage of fibrosis, for evaluating the true potential of this technique in the staging of fibrosis. In addition, a T1ρ MRI pulse sequence which enables T1ρ mapping data with reduced breathing artifacts is required. As such, in Allkemper et al.’s study respiratory triggering was used, however, it is difficult to control small motion displacement between different times of spin lock (TSLs) data. In other reported liver studies, poor breath holding or respiration induced displacement between different TSLs could be a substantial source of error [20]. Acquisition of entire T1ρ map data with different TSLs, corresponding to a single slice, in a single breath-hold can mitigate motion problems due to breathing.

In the current study, we have evaluated the potential of T1ρ MRI technique in the diagnosis of human patients with liver fibrosis. We implemented a spin locked MRI pulse sequence for T1ρ mapping of human liver in vivo on a 1.5 T clinical scanner and evaluated the feasibility of proton T1ρ relaxation mapping in detecting and quantifying changes due to liver fibrosis. Proposed pulse sequence enables acquisition of single 2D slice T1ρ MRI data with different TSLs in a single breath-hold. T1ρ values of patients were compared with histological staging and inflammation score of the liver in patients.

## Methods

Fourteen subjects, seven healthy (age = 27–65 years old) and seven patients (age = 40–70 years old) with liver fibrosis underwent MRI on 1.5 T clinical scanners (Siemens Medical Systems, Malvern, PA, USA). All the liver patients were diagnosed with chronic hepatitis C. The study protocol was approved by the Institutional Review Board of the institute and all subjects provided written informed consent. Subjects were positioned in the bore of the magnet, headfirst supine, with the body array coil placed superiorly and the vendor-supplied spine array coil located inferiorly. All the subjects were instructed to provide a gap of around 3 h between meal and MRI scan.

To minimize breathing related motion artifacts, MRI data were acquired during breath-hold. A gap of around 20 s was provided before starting next MRI acquisition. Imaging started with a tri-plane localizer followed be regular clinical anatomical imaging sequences, including T1- and T2-weighted imaging. For T1ρ imaging, an axial slice in the center of the liver was selected guided by anatomical T2 weighted images. T1ρ pulse sequence consisted of two parts: spin-lock pulse cluster and segmented turbo-flash readout with a spoiler for each part. T1ρ pulse clusters consist of 90°(+x) − SL(+y) − 180°(+y) − SL(−y) − 90°(−x) pulse [21], where SL represents spin lock. This is an optimized version of basic T1ρ pulse clusters [22] to account for B_0_ inhomogeneity. The SL pulse consists of spin lock amplitude, B1sl (= (γB_1_/2π) = ω_1_/2π) and spin lock duration (TSL). Parameter γ represent Gyromagnetic ratio, ω_1_ represents frequency. In order to retain maximum T1ρ weighting and minimizing the T_1_ recovery, centric encoding was implemented.

*T1ρ imaging protocol* T1ρ imaging was performed with spin lock duration (TSL) = 0, 10, 20, 30 ms, spin lock pulse amplitude B1sl = 500 Hz, TR/TE = 5.1/2.4 ms, flip angle = 10°, FOV = 300 × 300 mm^2^, matrix size = 128 × 128, slice thickness = 10 mm, number of shots = 1. The delay between successive T1ρ clusters was set to 2.5 s. The data corresponding to a single slice and four TSLs were acquired during one breath-hold period (scan time = ~12 s).

Before acquiring final data from subjects, the T1ρ imaging protocol was optimized in term of scan time and numbers of TSLs. Maximum TSL was limited by the scanner. Reproducibility studies were also performed on five healthy subjects. Two data sets in the same day MRI session (5 min gap) and different day MRI session were acquired for testing reproducibility of T1ρ mapping.

*Liver Histology* The biopsy specimens from each patient were obtained and fixed in a formalin solution and embedded in paraffin. Immunohistochemistry was performed to calculate the METAVIR score. Following METAVIR score [23] were used by Pathologists for staging liver fibrosis (F): 0 = no scarring; 1 = minimal scarring; 2 = scarring has occurred and is inside the areas of the liver including, blood vessels; 3 = bridging fibrosis (the fibrosis is spreading and connecting to other areas that contain fibrosis); 4 = cirrhosis or advanced scarring of the liver. Additionally, following grades, based upon METAVIR scoring [24], were used for determining inflammation activity (A) in liver biopsy specimens: 0 = no; 1 = mild; 2 = moderate; 3 = sever inflammation.

*Image Processing and Data Analysis* Liver tissue was manually segmented on T1ρ-weighted MRI image corresponding to TSL = 30 ms. The choice of T1ρ W image corresponding to longest TSL was based upon the observation that areas with any B_0_/B_1_ inhomogeneity artifacts are better visible on T1ρ W image corresponding to long TSL. Therefore, areas corresponding to these B_0_/B_1_ inhomogeneity artifacts can be easily avoided. Moreover, longest TSL in the current study was only 30 ms, so SNR was fairly good for segmentation. The T1ρ-weighted (T1ρ-W) data signal (S(TSL)) corresponding to four TSLs were fitted voxel-wise to mono-exponential decay expression, $S(TSL) = S\left( 0 \right)*exp( - TSL/T1\rho )$, for computing T1ρ values using an in-house written code in MATLAB (2007b). Coefficient of determination (R^2^) was used to determine quality of exponential function fit and voxels with R^2^ less than 0.8 were excluded from T1ρ map of liver. Mean and standard deviation (SD) of T1ρ values were computed for a single region of interest (ROI) on liver tissue, excluding any visible blood vessel voxels, from all the data. The size and location of ROI on T1ρ map was kept similar across all the subjects. T1ρ color maps of manually segmented liver sections were overlaid on baseline gray scale images (TSL = 0). Coefficient of variation (COV) for repeatability test was determined as ratio of SD to mean value of two measurements for each subject’s data. COV was calculated for the same ROI as mentioned above. Final COV was reported as average value of COVs for five subjects.

Student’s T-test was performed to compare T1ρ values between healthy and fibrotic livers. For studying correlation between T1ρ values vs stage of fibrosis, subject’s data were divided into four groups based upon the stage of fibrosis on the basis of histological information on patients. All healthy volunteer’s data were considered into group-0, subjects with stage-1 fibrosis in group-1, subjects with stage-2 in group-2 and subjects with stage-3 or 4 in group-3. Bar plots of liver T1ρ values were plotted against different groups. Correlation between T1ρ values of fibrotic liver and stage of fibrosis was computed. Relationship between T1ρ values of fibrotic liver and inflammation score was also studied.

## Results

**Fig. 1:**
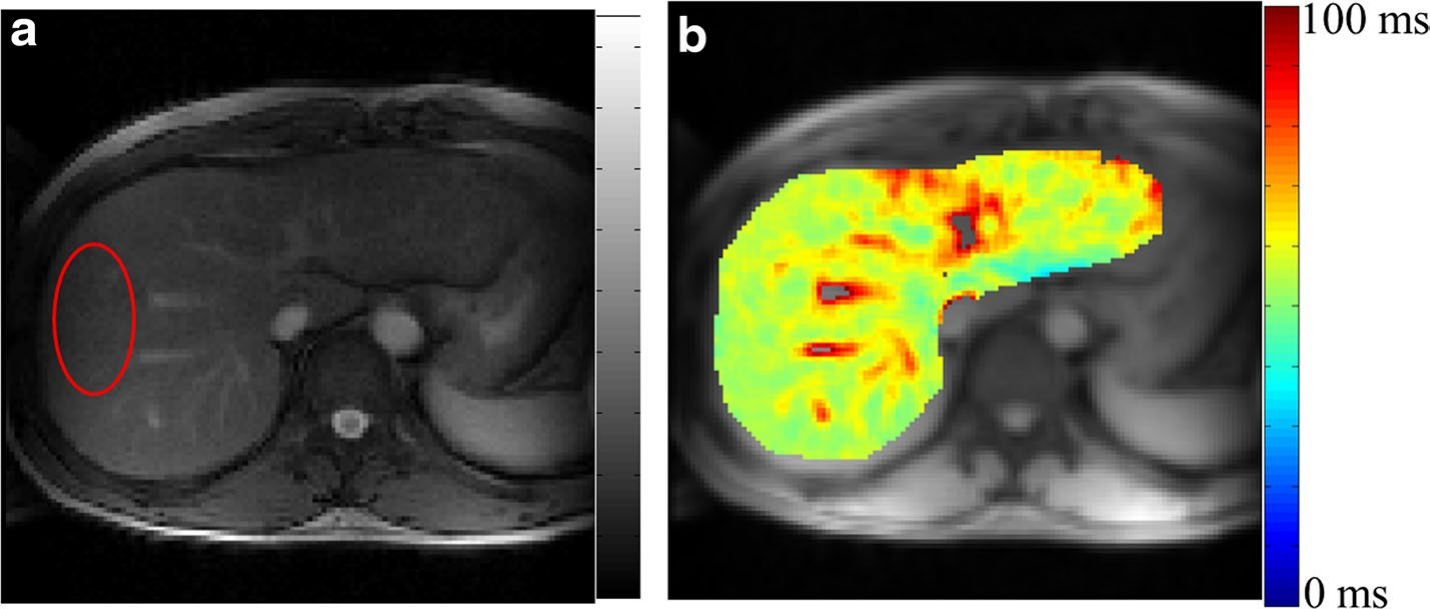
T1ρ MRI weighted image corresponding to TSL = 30 ms (**a**) and map (**b**) of healthy human liver. T1ρ map of segmented liver is color overlaid on anatomical image. T1ρ value in ROI marked on weighted image (**a**) is 55.6 ± 2.3 ms

Average T1ρ value along with inter subject SD in healthy liver was 51.04 ± 3.06 ms. T1ρ-W (TSL = 30 ms) image and T1ρ map from healthy liver are shown in Fig. 1. Average T1ρ values in ROI marked on liver in Fig. 1a is 55.6 ms. T1ρ values in voxels containing large blood vessels were set to zero based on poor R^2^ (<0.8) value. B_0_ and B_1_ field inhomogeneity artifacts appeared on T1ρ weighted images of some subjects, particularly on the edges close to heart. Usually these voxels exhibited poor fit and were excluded from the main analysis. While some of the voxels containing blood vessels can still be visualized as high T1ρ values compared to normal liver tissue and these might interfere with interpretation of results. To avoid this problem ROIs were carefully drawn in liver tissue, excluding any visible blood vessel voxel. The COV of T1ρ values between two repetitions in the same day session was 0.83 ± 0.8 % and in different day session was 5.4 ± 2.7 %.

In the current study, following fibrosis scores were observed: 0 for one patient; 1 for two patients; 2 for three patients; 3 for one patient and 4 for one patient. For healthy subject data fibrosis score were not available. In the current study, we assumed fibrosis score of zero for healthy subjects data. An inflammation score of 2 for one patient and 1 for remaining patients was observed.

**Fig. 2:**
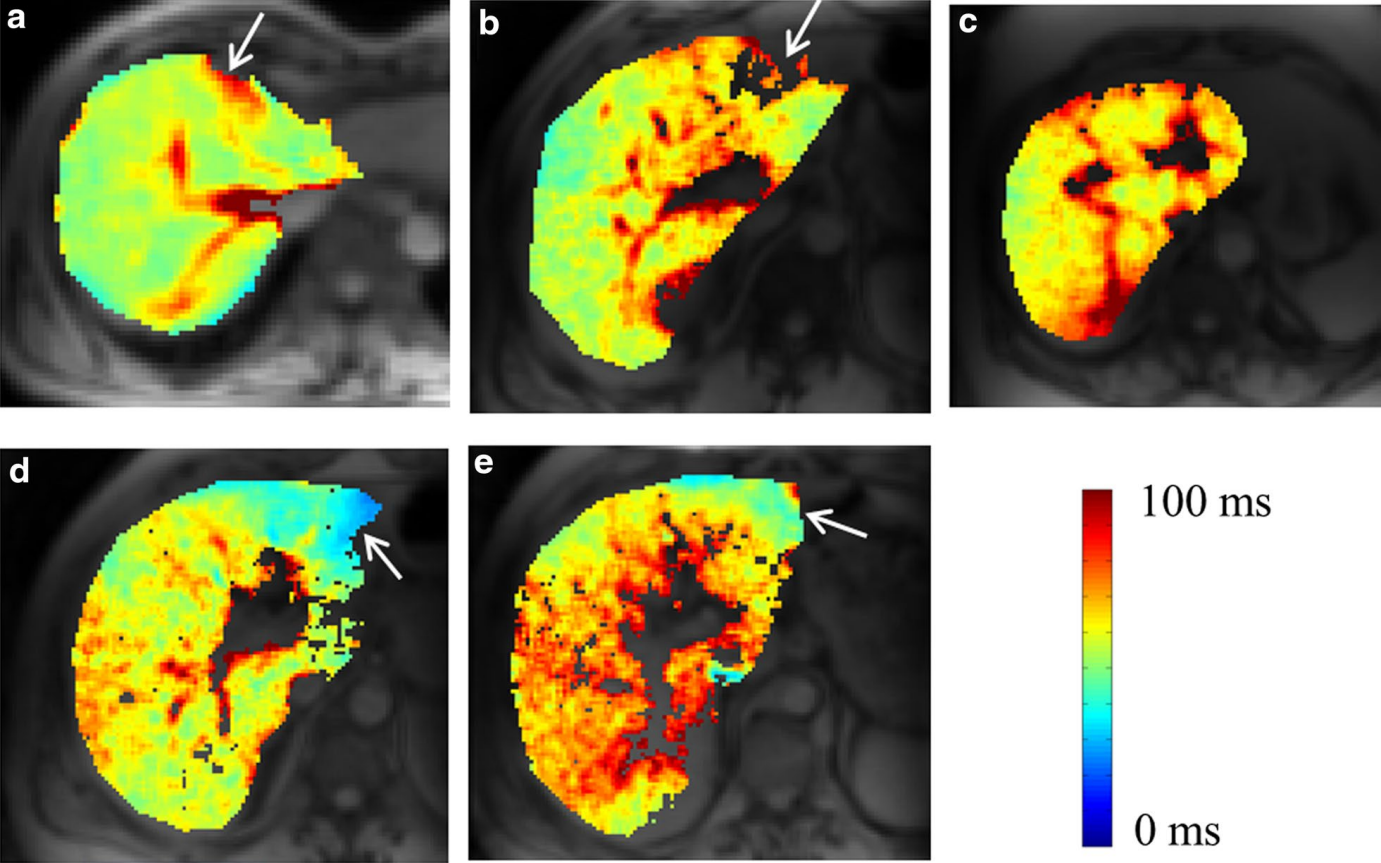
T1ρ maps of healthy human liver (**a**) and patients liver corresponding to fibrosis stage of 1, 2, 3 and 4 respectively. Maps of segmented liver are color overlaid on anatomical image. *White arrows* point to the areas with possible field inhomogeneity artifacts

T1ρ maps of another healthy subject (Fig. 2a) and patients with different fibrosis stage-1, 2, 3 and 4 are shown in Fig. 2. All of the patients had a same inflammation score (score = 1). Elevated T1ρ values in fibrotic livers are observed compared to the healthy liver. T1ρ maps of healthy subject, stage-1 and stage-2 (Fig. 2a–c) are homogeneous compared to stage-3 and 4 (Fig. 2d, e), excluding blood vessels. White arrow points to the areas having possible field inhomogeneity artifacts.

**Fig. 3:**
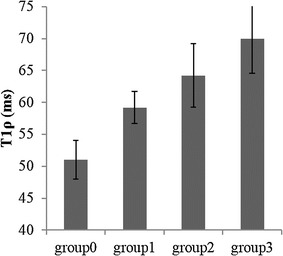
*Bar graph* of T1ρ values corresponding to different groups based upon fibrosis stage. Group-0 contains healthy subjects, group-1 contains stage-1 fibrosis, group-2 contains stage-2 fibrosis and group-3 contains stage-3 or 4 fibrosis. Fibrosis score are based upon METAVIR scale (0–4)

Bar plot of T1ρ values corresponding to different groups (fibrosis grades) is shown in Fig. 3. T1ρ values were 51.0 ± 3.0, 59.2 ± 2.5, 64.2 ± 4.5 and 69.9 ± 5.4 ms in healthy, stage-1, stage-2 and stage-3 and 4 respectively. T1ρ values in fibrotic liver were significantly (p < 0.05) higher compared to a healthy liver. Inter subject variations of T1ρ values are reflected by error bars. A high correlation (coefficient of correlation = 0.99) between T1ρ and fibrosis staging was observed. T1ρ values (mean ± SD) for all the patients along with fibrosis score and inflammation grades are shown in Table 1.

**Table 1:** T1ρ values (mean ± SD) for all the patients along with fibrosis score and inflammation grades based upon METAVIR score

Patient number	Fibrosis score	Inflammation grade	T1ρ (ms)
1	0	1	73.0 ± 3.7
2	1	1	57.4 ± 3.2
3	1	1	61.0 ± 3.5
4	2	2	60.7 ± 3.7
5	2	1	67.7 ± 5.4
6	3	1	66.1 ± 5.0
7	4	1	73.8 ± 6.4

**Fig. 4:**
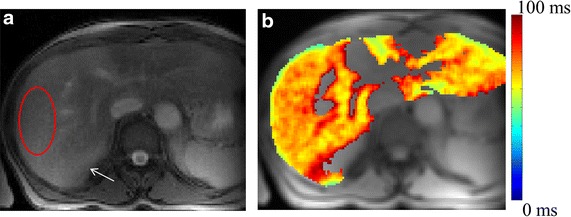
T1ρ weighted image corresponding to TSL = 30 ms (**a**) and map (**b**) of patient liver having stage-0 fibrosis and inflammation grade equal to 1. T1ρ map of segmented liver is color overlaid on anatomical image. T1ρ values in ROI marked on weighted image (**a**) is 73 ± 9 ms. *White arrow* points to liver tissue with partial volume to kidney

Figure 4 show T1ρ map of patient, having stage-0 fibrosis and inflammation grade equal to 1, based upon METAVIR scale. For this subject elevated T1ρ values were observed all across the liver compared to healthy subject’s liver. In fact T1ρ value for this subject was higher compared to average values in group-3 also. However, the overall T1ρ map of liver, except blood vessels, was homogeneous. During analysis, in stage-0 we have included all the healthy subjects and this subject’s data was excluded from analysis.

## Discussion

In the current study, the feasibility of single slice T_1ρ_ mapping of liver in a single breath-hold was demonstrated on 1.5 T clinical scanner and preliminary data from healthy and fibrotic human liver is presented along with histological results from fibrotic livers. Preliminary results from current studies have shown the potential of differentiating between healthy and fibrotic livers. These results are in agreements with recently reported T1ρ studies in liver [17, 18]. Previous reported studies have included data only from late stage fibrosis and healthy subjects. In the current study, data from healthy as well as fibrotic liver corresponding to different stage of fibrosis have been included. Preliminary results from current studies have also shown a high correlation between the stage of fibrosis and T_1ρ_ values. However, the number of subjects in different stages of fibrosis were small and therefore further studies on more subjects need to be performed for conforming the current observations. In the current study, inflammation score for all patients was equal to 1 except for one subject it was 2. For healthy subjects, it was assumed to be equal to 0. Increase in fluid level should increase T1ρ values and which was also reflected by higher T1ρ values in the patients data compared to healthy liver. However, this change in inflammation score is poorly correlated with an increase in fibrosis. This might be due to the fact that during liver fibrosis, other mechanisms such as change in ECM also take place.

Breathing induced motions, which could result in erroneous T1ρ mapping, were avoided by collecting T1ρ data corresponding to multiple TSLs in a single breath-hold period. Design and protocol of T1ρ pulse sequence used in the current study enabled collection of entire T1ρ data, required for generation of T1ρ map, in a single breath-hold period of ~12 s. T1ρ data corresponding to multiple slices can be collected in a similar manner during different breath-hold periods. In the current study, we have presented data corresponding to single slice. In most cases of chronic liver diseases causing fibrosis, such as viral and autoimmune hepatitis, as well as steatohepatitis, affect the liver in a relatively uniform way [25]. In the current study, all the patients were diagnosed with chronic hepatitis C. Therefore, the results of the current study should not be affected by choice of mismatch between biopsy location and imaging slice or a single ROI results. Therefore, results from a single slice of liver should be sufficient for the initial demonstration purpose. Moreover, it is feasible to apply the same approach to acquire data from multiple slices. In fact, we did collect T1ρ data corresponding to multiple slices (n = 8) for some subjects. This data was collected in multiple s, a single slice data (4 T1ρ W images) per breath hold was collected. Since multiple slice data was not collected for all the subjects, we have not included the results of multislice T_1ρ_ mapping in the current study.

Another, challenge in liver imaging is extensive vasculature of the liver parenchyma. Computation of T1ρ values in voxels containing vasculature is erroneous due to flow as well as the use of short TSLs. The majority of voxels in or containing large blood vasculature were removed based upon R^2^ values; however, voxels containing small vasculature were present in final T1ρ maps. T1ρ values in these voxels are not reliable and therefore these voxels should not be considered in the final analysis. To avoid this problem, we have presented T1ρ values from a small ROI excluding voxels with any visible vasculature.

T1ρ values were reproducible as demonstrated by small COV values for T1ρ in two different time experiments. Moreover, variations (SD) of ~3 ms (which is ~6 % of mean value) were observed among healthy subjects average T1ρ values.

In the current study, have used Pearson correlation coefficient for assessing correlation between T1ρ values and fibrosis score. Reported preliminary studies on T1ρ in liver have shown higher T1ρ values for fibrotic liver. For simplicity we have assumed a linear increase and that is the reason for use of Pearson correlation. As such, T1ρ values for stage-4 (74 ms) are higher compared to stage-3 (66 ms); however, due to only one subject for each of these two stages we have combined results of stage-3 and 4.

In the current study, we have scanned subjects over a wide age range. Aging results change in liver at both structural and functional level [26]. However, recently published study [17] have shown no relevant correlation between T1ρ values and age in liver.

Segmented turbo-flash readout has an advantage in terms of reduction in SAR deposition and fast imaging. However, flash readout can reduce T1ρ contrast, due to T_1_ recovery. In order to preserve maximum true T1ρ contrast, we used a centric encoding scheme in the current study and acquired only 128 lines during a single shot. This number was chosen based upon temporal resolution and T1ρ contrast preservation.

Depending on the tissue under consideration, correction of B_0_ and B_1_ field inhomogeneities and a proper combination of B1sl amplitude and SL duration is required for accurate computation of T1ρ map. Liver tissues have high field inhomogeneities, and automatic or interactive shimming does not work well in liver tissue. In recent years, significant attempts have been made in T1ρ technique implementation to minimize B_1_ and B_0_ inhomogeneities [21, 22]. Inclusion of a 180° pulse in T1ρ pulse cluster elevates SAR, although it minimizes the artifacts that can arise from B_0_ field inhomogeneities. In spite of use of this B_0_ and B_1_ field inhomogeneity compensation pulse cluster, some artifacts were observed in the voxels on liver particularly close to the lung. For reducing SAR accumulation, low SAR readout option has been used in the current study. There may be small errors associated with T1ρ estimation as we have used TSLs of only up to 30 ms.

## Conclusions

In conclusion, T1ρ mapping of human liver is feasible within SAR limits on 1.5 T clinical scanner. Proposed T1ρ pulse sequence design and protocol enabled the acquisition of entire T1ρ data of a single slice, corresponding to 4 TSLs, in a single breath-hold period and hence mitigated breathing motion related artifacts. Preliminary T1ρ MRI results suggest the potential of using T1ρ values in the diagnosis of liver fibrosis. Data acquisition from a large pool of patients, with different stages of fibrosis, is required before making any final conclusions on the potential use of T1ρ MRI for diagnosis and staging of liver fibrosis.
